# Molecular Principles of Fungal Pathogenesis

**DOI:** 10.3201/eid1210.060832

**Published:** 2006-10

**Authors:** Errol Reiss

**Affiliations:** * Centers for Disease Control and Prevention, Atlanta, Georgia, USA

**Keywords:** Molecular Principles of Fungal Pathogenesis, Joseph Heitman, book review, review

This book offers advanced treatment of a broad selection of topics in molecular medical mycology authored by leading investigators. It assumes a foundation of knowledge of mycotic pathogens and is suitable for the reader who is well-grounded in molecular microbiology. It is highly recommended for investigators planning to conduct medical mycology research. The book is divided into 5 sections: General Principles, Model Systems, Specific Pathogens, The Host, and Future Directions. Only selected highlights are described here because of space limitations.

The book reviews the development of transforming and gene-silencing methods for identifying virulence determinants. An overview of Candida albicans virulence underlines that molecular subtyping has elucidated 3 major clades, which differ in their potential for producing superficial versus deep-seated infection. The first step in pathogenesis is adherence to host tissues. The endothelial and epithelial specificity of members of the C. albicans Als family of adherence molecules is defined by the adherence profile of null mutants. The discovery through the genome sequence of C. albicans mating type locus and the delineation of the unique pathway of a parasexual cycle are discussed. Although the population is largely clonal and seems locked in a diploid state, the species has a demonstrated ability to undergo recombination.

The phylogenetic species concept has led to a better understanding of the lineage of pathogenic fungi, especially for the mitosporic fungi, which have no known sexual stage. The evolution of fungal species, shown by multilocus sequence typing, enabled construction of a phylogenetic tree of all known fungal pathogens with assignments to well-described families and orders.

Mechanisms of resistance to antifungal agents are discussed, including insights from genome sequence analysis and recent clinical observations such as the role of transcription factors in upregulating efflux pumps in the presence of antifungal agents or steroids. How environmental fungi have acquired their pathogenic potential for humans, even those whose immune function is intact, is a puzzle, but clues come from the interaction of fungi with soil-dwelling amebae. Fungi escape endocytosis by converting from yeast to hyphal forms; this not only conditions them for intracellular survival but also suggests how dimorphism may have originated. Transcriptional profiling using microarrays is a powerful tool for identifying genes expressed during mold-to-yeast morphogenesis and host-fungus interactions in infected tissue cultures and biofilms. Interspersed in the book are examples of exploiting this technology to discover key regulatory pathways.

No subject attracts more interest, yet is strewn with more pitfalls, than fungal vaccine development. Two major types of vaccine development are preventive vaccines and monoclonal antibody immunotherapy. Individual recombinant antigens have evoked mild to moderate protection. Interest in a potential attenuated live vaccine has been stimulated by the finding that targeted disruption of the Blastomyces dermatitidis gene, which encodes the surface adhesin BAD-1, renders the mutant avirulent.

Of the chapters on specific pathogens, the one covering virulence mechanisms in Coccidioides immitis is notable. It discusses application of molecular approaches to identify key proteins expressed during arthroconidial and spherule morphogenesis at each stage of the disease process and to dissect the corresponding interactions with the immune system.

